# Does Molimina Indicate Ovulation? Prospective Data in a Hormonally Documented Single-Cycle in Spontaneously Menstruating Women

**DOI:** 10.3390/ijerph15051016

**Published:** 2018-05-18

**Authors:** Jerilynn C. Prior, Chiaki Konishi, Christine L. Hitchcock, Elaine Kingwell, Patti Janssen, Anthony P. Cheung, Nichole Fairbrother, Azita Goshtasebi

**Affiliations:** 1 Centre for Menstrual Cycle and Ovulation Research, Vancouver, BC V5Z 1M9, Canada; chris@hitchcock.com (C.L.H.); elainejk@mail.ubc.ca (E.K.); patti.janssen@ubc.ca (P.J.); acheung@fertilitywithgrace.com (A.P.C.); azita.goshtasebi@ubc.ca (A.G.); 2 Division of Endocrinology, Department of Medicine, University of British Columbia, Vancouver, BC V5Z 1M9, Canada; 3 School of Population and Public Health, University of British Columbia; Vancouver, BC V6T 1Z3, Canada; 4 BC Women’s Health Research Institute, Vancouver, BC V6H 3N1, Canada; 5 Department of Educational and Counselling Psychology, McGill University, Montreal, QC H3A 0G4, Canada; chiaki.konishi@mcgill.ca; 6 Division of Neurology, Department of Medicine, University of British Columbia, Vancouver, BC V6T 2B5, Canada; 7 Division of Reproductive Endocrinology and Infertility, University of British Columbia, Vancouver, BC V6T 2A1, Canada; 8 Grace Fertility Centre, Vancouver, BC V5Z 1G1, Canada; 9 Department of Psychiatry, University of British Columbia, Vancouver, BC V6T 2A1, Canada; nicholef@uvic.ca

**Keywords:** menstrual cycle, ovulation, molimina, pregnanediol, axillary breast tenderness, subclinical ovulatory disturbances, premenstrual symptoms, osteoporosis, Menstrual Cycle Diary

## Abstract

Approximately 33% of normal-length (21–35 days) cycles have subclinical ovulatory disturbances and lack sufficient progesterone, although their normal length ensures enough estrogen. Subclinical ovulatory disturbances are related to significant premenopausal spine bone loss (−0.86%/year). Molimina, non-distressing premenstrual experiences, may detect ovulation within normal-length cycles. This prospective study assessed the relationship between molimina and ovulation. After 1-cycle of daily diary and first morning urine collections, women answered the Molimina Question (MQ): “*Can you tell by the way you feel that your period is coming?*” and were invited to share (a) predictive premenstrual experience(s). A 3-fold increase in follicular-luteal pregnanediol levels confirmed ovulation. In 610 spontaneously menstruating women (not on hormonal contraception, mean age 31.5 ± 5.3, menarche age 12.7 ± 1.5, cycle length [CL] 29 days, MQ positive in 89%), reported premenstrual experiences which included negative moods (62%), cramps (48%), bloating (39%), and front (26%) or axillary (25%) breast tenderness. Of 432 women with pregnanediol-documented cycles, 398 (92%) were ovulatory (CL: 29 ± 5) and 34 (8%) had ovulatory disturbances (CL: 32 ± 14). Women with/without ovulatory cycles were similar in parity, body mass index, smoking, dietary restraint and the MQ; ovulatory-disturbed cycles were longer. Molimina did not confirm ovulation. A non-invasive, inexpensive ovulation indicator is needed to prevent osteoporosis.

## 1. Introduction

Traditionally, estrogen/estradiol is understood to preserve premenopausal areal bone mineral density (BMD) and thus prevent osteoporosis [1]. Hypothalamic amenorrhea is strongly associated with rapid bone loss, low bone density, and fracture; these risks at the population-level can even be seen with longer cycle lengths within the normal range of 21–35 days [2,3]. In addition, estradiol-sufficient, normal-length cycles are not always ovulatory and may not produce progesterone levels that are sufficient to counterbalance and complement the effects of estradiol [4]. Incident subclinical ovulatory disturbances (anovulation and short luteal phases within cycles that are predictable and of a regular-length) are common. They occur across one year for almost two-thirds of normal-weight, non-smoking women, initially proven normally cycling/ovulatory in two consecutive cycles; subclinical ovulatory disturbances were related to significant bone loss [5]. Based on a large population study of spontaneously cycling women with predictable, normal cycle lengths, anovulation by a cycle-timed serum progesterone level of ≤9.5 nmol/L occurred for more than 33% of women [6]. In a meta-analysis of prospective studies that tracked cycles, ovulation and BMD changes in premenopausal women, menstrual cycles with ovulatory disturbances were more prevalent than the group mean and had a weighted mean difference in spinal BMD change of −0.86%/year versus those with fewer subclinical ovulatory disturbances [7].

Spinal BMD loss of almost one percent a year, if subclinical ovulatory disturbances persist over the lifecycle of 30–40 menstruating years, is likely to put women at risk of osteoporosis and fragility fractures [8]. Subclinical ovulatory disturbances, like hypothalamic amenorrhea, are likely to occur because of various or combined “stressors” including eating too few calories to meet energy requirements, emotional distress, physical, psychological or sexual abuse or illnesses [9]. If these silent ovulatory disturbances are detected, bone loss can be prevented, and bone can be regained by treatment with “replacement” cyclic progesterone therapy [10]. Cognitive behavioral therapy has also been shown to allow reproductive recovery for hypothalamic amenorrhea [11,12].

To prevent later-life osteoporosis for women in the population, however, since less than 5% of women experience oligo/amenorrhea during one year [13], we need a way to identify subclinical ovulatory disturbances that pose an overall higher risk for osteoporosis. Since ovulatory disturbances are reversible [14] and treatable [10], to prevent subclinical premenopausal bone loss, we must detect silent ovulatory disturbances.

Prospective one-year data show that a single incident anovulatory cycle or two short luteal phase cycles per year were associated with loss of 4–6% annual cancellous volumetric spinal BMD [5]. Therefore, to detect relevant subclinical ovulatory disturbances, assessment of ovulation needs to occur for all or most cycles. Other than for fertility testing, the current cycle-timed laboratory tests that are available (such as the urinary luteinizing hormone [LH] midcycle peak) are too invasive, difficult to organize, and expensive when considering its practical use in cycle-by-cycle testing over longer durations.

Molimina is an older medical term which refers to the normal, non-bothersome changes in experience that women may perceive before menstruation (premenstrually). Molimina is reported to occur *only* in ovulatory cycles [15] and may involve physical changes such as fluid retention [16], breast tenderness, or negative moods [17]. Breast tenderness is of two indicative types: front or sub-areolar tenderness (associated with higher estradiol levels) or axillary or high lateral breast tenderness with the rest of the breast being asymptomatic (associated with luteal levels of progesterone and ovulatory cycles) [18]. Molimina has further been shown to be reliably *absent* from anovulatory cycles in a clinical study of 61 regularly cycling, normal-weight women seeking care for symptomatic hirsutism [18]. Thus, although there were two past studies seeking to validate the molimina-ovulation relationship, they both had small sample sizes and methodological limitations [6,7].

The purpose of the present single-cycle study was to confirm the relationship between molimina and an ovulatory cycle as hormonally documented using validated urinary pregnanediol (PdG) collection, analysis [19] and evaluation methods [20]. We expected that an ovulatory cycle (as confirmed by a 3-fold follicular-to-luteal phase increase in pregnanediol [PdG]) would be associated with molimina by spontaneously reported axillary breast tenderness.

## 2. Methods

### 2.1. Participants

Women were eligible to participate if they were spontaneously menstruating and had bled in the last three months, between ages 20–40 years and, in the last six months, had not been on any form of hormonal contraception. Over 18 months, using multiple recruitment methods as previously described [21], we enrolled a convenience sample of 610 eligible women. They were recruited from the Metro Vancouver (BC, Canada) region, a geographically large urban/suburban area with a population of about two million. All women provided written informed consent to a study approved by the University of British Columbia Clinical Research Ethics Board (#C06-0390).

Women were taught to record the Menstrual Cycle Diary© [22] through viewing an instructional YouTube video https://www.youtube.com/watch?v=6K9LB6afKxE and were requested to record a wide variety of experiences every night before bed. They started diary records on the first day of flow and continued until the second day of the next menstruation. They were provided with two special “paper” diaries suitable for digital scanning. Digital diary data were subsequently cross-checked against the originals; corrected data were subsequently entered into a database for integration with other participant-specific information.

Women were unpaid volunteers. However, all women were provided with feedback about their own menstrual cycle hormonal information and ovulatory status.

### 2.2. Flow of Participants through the Study

Women were scheduled for two visits. In their first visit, they completed a comprehensive interviewer-administered questionnaire including socio-demographic, physical, racial/cultural, reproductive and medical and lifestyle characteristics; and a modified version of the questionnaire that was used in the baseline phase of the Canadian Multicentre Osteoporosis Study (with permission) [23]. The Molimina Question (see Section 2.3) was embedded within this modified questionnaire. Participants were also instructed for their diary and urine specimen collection and had their height and weight measured for body mass index (BMI) in the m^2^/kg calculation. For the second visit, after the end of the study cycle, they were asked to bring in their frozen urine specimens and completed diary sheets. The Molimina Question was asked again; this second response and its volunteered indicative experience were used as the primary outcome of the analysis.

### 2.3. Molimina Documentation

The Molimina Question (MQ) was first validated in consecutive clinical series of 20–35 year old women with regular menstrual cycles, but whose reason for seeking medical attention was due to hirsutism (unwanted facial hair) [18]. In an interviewer-administered questionnaire, they were systematically asked: “Can you tell by the way you feel that your period is coming?” The response was scored as “always” (4), “usually” (3), “about half the time” (2), “rarely” (1), or “never” (0). The responses of (4) and (3) were categorized as ‘positive’, and responses of (2), (1) or (0) were categorized as ‘negative’”. The majority of women (46 of 61) felt they could not tell that their period was coming (responses of 0–2); however, 15 felt that they could feel that their period would soon start [3,4]. In that or the next cycle, cycle-timed serum progesterone (a serum level of 16 nmol/L was used as evidence for ovulation) was obtained on or about day 22 and confirmed to be at least three days before the next flow. Analysis showed that a negative Molimina Question had a positive predictive value for anovulation of 96%, a negative predictive value of 93%, a sensitivity of 88% and specificity of 98% based on a serum progesterone level of 16 nmol/L for ovulatory status [18]. In a different cohort, we further assessed the MQ for reliability in ~450 women, tested twice four weeks apart. Analysis using Cronbach’s Alpha showed that there was acceptable reliability between two questions within the same woman (α = 0.864).

The MQ was thus created by the first author (JCP) to be included in the Canadian Multicentre Osteoporosis Study questionnaire [23]. The MQ was followed by a request to spontaneously describe or report their most indicative premenstrual experience(s). These moliminal experiences, obtained after repeated requests but without specific prompting, were grouped by the interviewer into the following categories: cramps, increased appetite, bloating/fluid retention, breast tenderness (front or axillary or both), negative moods, headaches, breast swelling and acne. A participant could provide responses in more than one category.

### 2.4. Hormonal Cycle Documentation

Women were taught to collect the first morning overnight urines in a specially designed 7 mL container of bisphenol A- and hormone-free polypropylene with a small sponge embedded in the screw-top lid. They were asked to collect two specimens a week starting at cycle day eight. They were provided with printed labels including their unique study number and instructed to write the date and attach the label to a filled urine container before placing it in a provided 2 L insulated carry bag with a zipper closure and storing it in their home freezer (for a maximum of six weeks).

The returned urine samples were stored in the laboratory’s −70 degree research freezer for 1–20 months before being shipped via courier on dry ice to the measuring laboratory [19]. Urinary PdG, estrone conjugates (E1C) and specific gravity (to assess urine concentration) were measured in each first-morning urine by previously validated methods [19]. The primary ovulation-assessment method (3-fold follicular-luteal PdG increase) was validated for non-daily samples [10]. If there were problems with the primary method, ovulation was confirmed by the method of Baird [24], identifying the day on which there was a cross in data as the estrone conjugate (E1C) levels decreased (following the midcycle estrogen peak) and PdG levels increased into the luteal phase.

### 2.5. Statistical Analysis

We first characterized the cycle as ovulatory versus ovulatory-disturbed (meaning either anovulatory or assumed ovulatory but with a short or insufficient luteal phase). The cohort was described by socio-demographic, physical and reproductive characteristics [23]. We also compared the responses on the first visit (pre-cycle) versus the second visit (post-cycle) MQ. Subsequently, we described demographic and reproductive differences, and the proportion of positive responses to the second visit MQ, between those with ovulatory versus ovulatory-disturbed cycles. Lastly, we compared the volunteered experiences (descriptively) by ovulatory status. All analyses were completed using SPSS software (IBM Corp. Released 2016. IBM SPSS Statistics for Windows, Version 24.0. IBM Corp, Armonk, NY, USA).

## 3. Results

**Figure 1:**
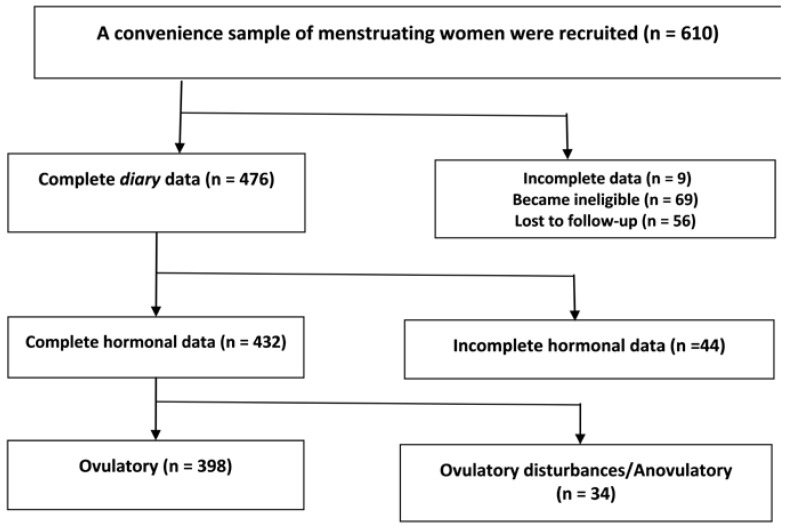
Flow diagram of community-dwelling women recruited to the Molimina and Ovulation Study who, at study entry, were spontaneously cycling, not using hormonal contraception and aged 20–40 years.

Of the 610 enrolled women, 476 remained eligible and completed the diary; evaluable hormonal data were collected by 432 of these women. The flowchart of participant recruitment through to participation in this study is shown in Figure 1. Of the 69 women who became ineligible, 36 began hormonal contraception. Thirty-three of those who became ineligible did so because they became pregnant within that cycle.

Characteristics of the women for whom molimina could be compared with ovulatory status (*n* = 432) are shown in Table 1 alongside the characteristics of the 610 women in the whole study cohort. The 432 women with adequate hormonal data differed from those without sufficient data (*n* = 179); those who did not complete the study were less educated (*p* = 0.011) and younger (*p* = 0.005), but their reproductive characteristics did not differ (data not shown).

On the first presentation of the MQ, eighty-nine percent (89%) of the 610 women in the cohort believed that they could tell by the way they felt that their menstruation would soon start (MQ positive = 3–4). The responses of those with hormonal data (*n* = 432) to the first MQ differed significantly from their responses to the second MQ by cross-tabulation (*p* = 0.001). In general, those who said that they could always tell, became less certain how frequently they experienced molimina. The opposite change occurred for those who initially reported that they could never tell who now said that sometimes they could tell by the way they felt that their period was coming.

The urinary hormone analysis revealed that 398 women met the criteria for a normally ovulatory cycle; the remaining 34 women with hormonal data had an “ovulatory-disturbed cycle” (meaning anovulation, short luteal phase or luteal insufficiency). Women with ovulatory disturbances (Table 1) were slightly but significantly younger, had experienced menarche at an older age, and had longer cycles compared with women with normal ovulatory cycles. However, these two groups did not differ in BMI, education or employment, and a similar proportion of women in each of the ovulatory status groups offered a positive response to the second Molimina Question; this included 89% of women with an ovulatory and 97% with an ovulation-disturbed cycle.

**Figure 2:**
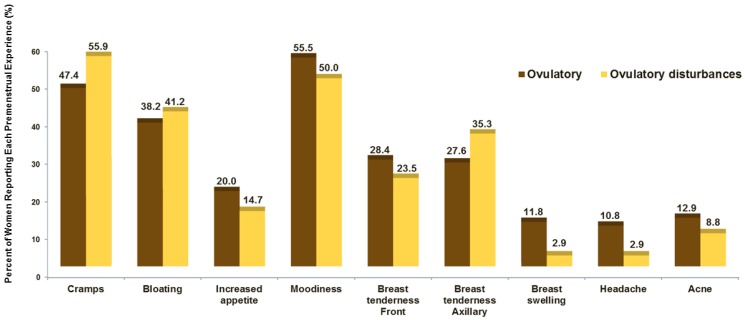
In the Molimina study the percentage of women reporting each premenstrual experience by ovulatory status group.

The request to volunteer a key experience that validated their MQ responses accrued a wide variety of experience changes (more than one for most women) as shown in Figure 2. This bar graph shows the percentage of women in the two ovulatory status groups who spontaneously mentioned a given premenstrual experience. About half of both ovulatory status groups voluntarily reported that cramps and negative moods (“moodiness”) were important premenstrual experiences. There were no significant differences between the two groups by ovulatory status, even for breast swelling that tended to be less frequent in women with ovulatory-disturbed cycles.

## 4. Discussion

This single-cycle study using the Molimina Question in a large urban convenience cohort of spontaneously cycling women between ages 20–40 years did not find that molimina was indicative of an ovulatory menstrual cycle, although we used a validated standard for evidence of ovulation (a 3-fold follicular to luteal increase in urinary pregnanediol levels) [20]. We found that only eight percent of these women experienced anovulatory or ovulation-disturbed cycles by this method. This is a significantly lower proportion than that expected, based on the single-cycle anovulation prevalence of 37% that was observed in a population-based sample of more than 3000 women using a cycle-timed serum progesterone level [6] above a validated threshold of 9.54 nmol/L) [25]. It is also significantly less than several other prospective studies [5,26,27] that used a validated quantitative basal temperature method [28]. Studies in other convenience samples that used similar urinary hormonal levels found similarly low proportions of anovulatory cycles [29,30], as observed in our cohort. Thus, there may be differences in the sensitivity of ovulatory criteria using cycle-timed serum progesterone levels and the quantitative basal temperature versus a 3-fold increase in urinary PdG levels from the follicular to the luteal phase.

The low proportions of apparent anovulation/ovulation-disturbed cycles compared with those that were ovulatory in our study did not allow us to calculate the valid estimates of the sensitivity and specificity of the Molimina Question [31]. In testing performed before we became aware of that statistical limitation, however, we found that the presence of spontaneously reported premenstrual *axillary* breast tenderness without front-of-the-breast tenderness was highly insensitive for ovulatory status (8.8% [95% CI 6.3, 12.0%]). However, axillary breast tenderness was highly specific for an ovulatory cycle (100% [95% CI 79, 100%]). This observation requires further investigation.

Over half of the women reported dysmenorrhea (cramps) as a premenstrual experience that suggested that their period would soon start. Since cramps normally precede flow only by hours or perhaps a day and do not appear to differ between ovulatory and anovulatory cycles, this suggests that “molimina” that includes cramps is unlikely to indicate an ovulatory cycle. Also, because in North America there is a strong cultural expectation that women become moody premenstrually, with frustration, depression or anxiety, and over half of the women, regardless of ovulatory status reported “moodiness,” there is further evidence that cultural expectations may play a role in the responses we elicited.

One limitation of this study may be that we studied a single cycle; women might well be referring to a previous set of menstrual cycle-related experiences (of unknown duration) when answering these two Molimina Questions. Also, it is unlikely that these women had previously specifically observed or recorded their cycle-related experience changes. Another limitation is that this was a fairly highly educated sample, in part because recruitment included an institution-wide email invitation to employees of a large urban academic medical center [21]. Participant responses *did* significantly change in the second MQ after they had recorded a cycle’s daily diary data. There may also have been a large (unmeasured) component of cultural expectation related to the past local publicity around the “premenstrual syndrome” or “PMS” that could have influenced women’s responses, especially in reporting “moodiness” which over half of the women did. In fact, almost all younger women (97%) with an ovulation-disturbed cycle reported that they usually or always could tell by the way they felt that their period was coming. Perhaps the notion of “molimina,” that originated when Medicine was very much a pattern-based rather than a science-focused discipline is simply not valid.

On the other hand, this study also had several strengths. It was a large study in an unscreened (no exclusion criteria except ovarian hormone-based contraception or therapy and not having menstruation) community-dwelling population of young adult women between ages 20–40 years. We used a validated, non-invasive method for documenting evidence of ovulation. In addition, we provided information on the specific menstrual cycle and health education. During a normal menstrual cycle, peak estradiol and progesterone levels increase by 240 and 1400 percent, respectively [32], as did various physiological functions such as the core temperature [33], bone resorption and formation biomarkers [34], caloric intakes [35] and likely QT intervals [36]. Given these hormonal and physiological changes are normal for ovulatory cycles, it would be surprising if women’s experiences and perceptions did not change.

## 5. Conclusions

Self-reported molimina, as measured by the standardized Molimina Question followed by volunteered specific premenstrual experience changes, was not an accurate indicator of ovulation-disturbed cycles (anovulatory or luteal phase disturbances) among women tested using a 3-fold increase in PdG from the follicular to the luteal phase as evidence for ovulation. Therefore, other than cycle-timed specific hormonal testing, we currently have no way of knowing whether a cycle that is regular and of normal length, is ovulatory. Given the high prevalence (37%) of subclinical ovulatory disturbances in the population and their association with negative changes in bone mineral density (−0.86% of spinal bone/year) and with likely risks for later-life fragility fractures (based on evidence that progesterone increases women’s bone formation and accounts for 20% of the variance in cancellous spinal bone change), other non-invasive, inexpensive and convenient methods for detecting the ovulatory status of regular, normal-length cycles are urgently needed.

## Figures and Tables

**Table 1:** The demographic, physical, socio-cultural and reproductive characteristics of women in the entire convenience cohort (on the left), and the women in the ovulatory and ovulatory disturbed subgroups. Statistical comparisons are of the two groups with hormonal data (*N* = 432) with significant *p* values in bold.

Characteristics	Total Cohort (*N*: 610)	Women with Hormonal Data (*N*: 432)		
		Ovulatory *N*: 398	Ovulatory Disturbances *N*: 34	*p* Value ^1^
Age (years) Mean (SD)	31.5 (5.3)	32.1 (5.3)	29.4 (5.2)	**0.005**
BMI (kg/m^2^) Mean (SD)	24.4 (4.7)	24.3 (4.4)	23.8 (6.2)	0.558
Education: *N* (%)				0.215
High-school or less	36 (5.9)	18 (4.5)	4 (11.8)	
University certificate or less	143 (23.4)	78 (19.6)	7 (20.6)	
University degree	431 (70.7)	302 (75.9)	23 (67.6)	
Employment: *N* (%)				0.964
Full-time	360 (59)	237 (59.5)	20 (58.8)	
Part-time	97 (15.9)	68 (17.1)	5 (14.7)	
Student	100 (16.4)	62 (15.6)	7 (20.6)	
Other	53 (8.7)	31 (7.8)	2 (5.9)	
Age at menarche (years) Mean (SD)	12.7 (1.5)	12.7 (1.4)	13.3 (1.5)	**0.012**
Cycle length (days) Mean (SD)	29.3 (6.1)	29.3 (4.7)	31.8 (14)	**0.015**
Molimina Question (3 = usually and 4 = always)	545 (89.3)	353 (88.7)	33 (97.1)	0.682

^1^ Comparison between the ovulatory and ovulatory-disturbed women based on the *t*-test or Chi-Squared test as appropriate.
